# Morphological Profile of Facial Acne Scars and Their Correlation With Body Image Concern and Scar-Specific Quality of Life: A Cross-Sectional Study From Rural India

**DOI:** 10.7759/cureus.110672

**Published:** 2026-06-11

**Authors:** Jayaprakash M H, Mukunda R Swaroop, Vinay H R, Yogesh Devaraj, Rahul H V, Jithin Jayaraj, Harshavardhan M, Nisarga Yoganand, Esha Nobbay, Divyasree R

**Affiliations:** 1 Dermatology, Adichunchanagiri Institute of Medical Sciences, Adichunchanagiri University, B.G. Nagara, IND; 2 Dermatology, Akash Institute of Medical Sciences, Bengaluru, IND; 3 Psychiatry, Siddaganga Medical College and Research Institute, Tumkur, IND; 4 Psychiatry, Adichunchanagiri Institute of Medical Sciences, Adichunchanagiri University, B.G. Nagara, IND

**Keywords:** acne, acne medication, acne scars, atrophic acne scars, body image concern, fasqol, goodman–baron grading, quality of life, recalcitrant acne, scarring acne

## Abstract

Introduction

Acne scarring represents a permanent structural sequela of acne vulgaris and is predominantly characterized by atrophic morphological patterns. Beyond cosmetic disfigurement, facial acne scars may contribute to significant psychosocial distress, including body image disturbance and quality-of-life impairment. Limited data are available correlating scar morphology with body image concerns and scar-specific quality-of-life impairment in the Indian population.

Objectives

The objectives of this study are to examine the morphological profile of facial acne scars, evaluate the body image concerns and quality of life among patients with acne scars, and determine the correlation of facial acne scarring with body image concerns and quality-of-life impairment.

Materials and methods

This cross-sectional observational study was conducted in the Department of Dermatology, Venereology and Leprosy, Adichunchanagiri Hospital and Research Centre, Karnataka, over a period of 18 months. It included 150 patients of either gender, aged 18-40 years, with facial acne scars. Morphological assessment was performed under standardized illumination. Scar severity was graded using the Goodman and Baron qualitative grading system and the Self-assessment of Clinical Acne-Related Scars (SCARS) questionnaire. The Body Image Concern Inventory (BICI) and Facial Acne Scar Quality of Life (FASQoL) questionnaires were used for fulfilling the respective objective. Statistical analysis was performed using Epi Info software. Correlations were assessed using Spearman’s rank correlation coefficient. A p-value of < 0.05 was considered statistically significant.

Results

The majority of participants (63.3%; n=95) were aged 21-25 years, and 69.3% (n=104) were female. Icepick scars were the most common morphological type at 78.7% (n=118), followed by boxcar scars at 61.3% (n=92). Clinician-based grading revealed that 72.6% (n=109) of participants had moderate to severe acne scarring. Based on a SCARS assessment, 36.0% (n=54) had mild, 30.0% (n=45) had moderate, and 15.3% (n=23) had severe/very severe scarring. Mean BICI scores increased significantly across SCARS severity categories (p<0.001). The mean FASQoL score was 6.79 ± 7.11, with higher scores observed in severe scarring (p<0.001). Moderate positive correlations were observed between SCARS and BICI (rₛ=0.419, p<0.001), SCARS and FASQoL (rₛ=0.500, p<0.001), Goodman-Baron grade and FASQoL (rₛ=0.448, p<0.001), and Goodman-Baron grade and BICI (rₛ=0.367, p<0.001).

Conclusion

Body image concern is evident across domains of dissatisfaction with appearance, preoccupation with perceived flaws, and mirror-checking behaviors. Scar-specific quality-of-life impairment manifests as emotional distress, social self-consciousness, and reduced interpersonal confidence. Patient-perceived severity demonstrates a particularly strong relationship with psychosocial burden, underscoring the importance of integrating clinician-based grading with validated patient-reported outcome measures for comprehensive assessment and patient-centered management.

## Introduction

Acne vulgaris is a chronic inflammatory disorder of the pilosebaceous unit characterized by comedones, papules, pustules, nodules, and, in severe cases, cysts, predominantly affecting adolescents and young adults, with peak incidence during the second and third decades of life [1,2]. In clinical practice, however, acne frequently follows a prolonged course with relapses and lasting sequelae, leading to scarring, which is the common and permanent sequela [3]. The chronicity of the disease, visibility of lesions, and need for long-term management make it a significant medical and psychosocial concern rather than a purely cosmetic disorder [4].

Facial involvement is particularly important due to constant visibility and social exposure [5]. In routine dermatology practice, it is often the residual scars, rather than active acne lesions, that prompt patients to seek further medical attention, underscoring their long-term clinical relevance [6].

The development of acne scars reflects an imbalance in the normal wound-healing process following inflammation, as a result of either loss of net collagen or deposition of excess collagen. Based on this, scars are broadly classified into atrophic and hypertrophic types [7,8].

Facial acne scars have a substantial psychosocial impact owing to their permanence and high visibility, and individuals might experience significant emotional as well as psychiatric distress, including embarrassment, self-consciousness, and reduced confidence in social interactions, leading to overall reduced quality of life [9,10]. In clinical settings, patients frequently report avoidance of social situations due to acne scars extending beyond physical disfigurement, which explains the perception of their body image [11,12]. Acne scarring represents a substantial clinical and psychosocial burden worldwide and is frequently encountered in dermatology practice. Indian studies have similarly highlighted the significant burden of acne scarring among young adults, yet comprehensive data evaluating its psychosocial impact and patient-perceived severity remain limited in the Indian population [13,14]. The development of tools, such as Self-assessment of Clinical Acne-Related Scars (SCARS) and Facial Acne Scar Quality of Life (FASQoL), has enabled a structured evaluation of scar burden [15].

The present study was undertaken to evaluate acne scar morphology and its association with body image disturbance and quality of life impairment using validated patient-reported outcome measures in a predominantly rural Indian population, a group that remains relatively underrepresented in acne scar research. Differences in access to dermatologic care, treatment-seeking behavior, and sociocultural perceptions related to facial appearance may influence the psychosocial burden associated with acne scarring in such populations.

## Materials and methods

This cross-sectional observational study was conducted in the Department of Dermatology, Venereology and Leprosy, Adichunchanagiri Hospital and Research Centre, B.G. Nagara, Karnataka. The study was carried out on an outpatient department (OPD) basis, catering predominantly to a rural population from April 2024 to September 2025. Ethical approval was obtained from the Institutional Ethics Committee prior to commencement of the study (IEC approval number: AIMS/IEC/032/2024).

Sample size was estimated using the standard formula for cross-sectional studies, $n = \frac{Z^{2} \times p \times (1-p)}{d^{2}}$, assuming a 95% confidence interval, 47% expected prevalence of acne scarring based on previously published literature [16], and 8% allowable error. The calculated minimum sample size was approximately 149 participants, which was rounded to 150 participants. A consecutive sampling technique was employed, wherein all eligible patients attending the dermatology outpatient department during the study period and fulfilling the inclusion criteria were recruited until the required sample size was achieved.

Inclusion criteria

Patients aged 18-40 years, with facial acne scars, who were willing to participate and provide written informed consent were included in the study.

Exclusion criteria

Patients who had received laser therapy, energy-based devices, or other procedural treatments for acne scars within the preceding six months were excluded. Individuals with previously diagnosed major psychiatric illness based on documented medical history or self-report were excluded. Patients with acne scars associated with other dermatological disorders affecting the face were also excluded from the study.

Procedure of the study

Written informed consent was obtained from all participants fulfilling the inclusion and exclusion criteria. A structured proforma was used to collect the required demographic and clinical details. Sociodemographic information, including age, sex, occupation, and address, was recorded along with relevant family history. Clinical history included age of onset of acne, duration, site distribution, types of lesions, treatment history based on participant recall, aggravating and relieving factors, and seasonal variation. A detailed facial examination was performed under standardized white light illumination by dermatologists. Patients with active acne lesions were not excluded if clinically significant facial acne scarring was present.

Acne scars were evaluated for morphology, including icepick, boxcar, rolling, and hypertrophic scars. Site distribution involving the cheeks, forehead, chin, nose, mandibular area, and upper lip was documented. The pattern of scarring was also assessed as either single or mixed morphology.

Severity assessment was performed using both clinician-based and patient-reported measures. The Goodman and Baron qualitative acne scar grading system was used for a clinician-based assessment of scar severity. Grade I represented macular scarring without visible contour deformity. Grade II included mild atrophic or hypertrophic scars that could be concealed with makeup or facial hair. Grade III represented moderate scars visible at social distances that could be flattened by manual stretching of the skin, while Grade IV included severe scars that were not easily corrected by stretching [8].

The SCARS questionnaire was used for patient-reported assessment of acne scar severity. The questionnaire consists of five items, each scored from 0 to 4, with total scores ranging from 0 to 20. Higher scores indicate greater perceived severity of acne scarring. Total scores were interpreted as clear/almost clear (0-2), mild (3-6), moderate (7-10), and severe/very severe scarring (11-20). This questionnaire is a validated patient-reported outcome measure developed specifically for the assessment of acne scar severity and has demonstrated acceptable validity and reliability in previous studies [15].

The Body Image Concern Inventory (BICI) was used to assess appearance-related dysmorphic concern and body image dissatisfaction. The questionnaire consists of 19 items scored on a 5-point Likert scale ranging from 1 to 5. Higher total scores indicate greater body image concern and appearance-related distress. The BICI is a validated instrument designed to assess body image dissatisfaction and appearance-related concerns and has demonstrated good psychometric properties, including validity and reliability, in previous studies [12].

The FASQoL questionnaire was used to assess scar-specific quality-of-life impairment across emotional and social domains. The questionnaire consists of 10 items scored on a 5-point Likert scale. The FASQoL questionnaire is a validated acne scar-specific quality-of-life instrument that has demonstrated satisfactory validity and reliability for assessing the psychosocial burden associated with facial acne scarring [5].

Statistical analysis

Data were entered into Microsoft Excel (Microsoft Corporation, Redmond, WA, US) and analyzed using Epi Info™ software (Centers for Disease Control and Prevention, Atlanta, USA). Continuous variables were expressed as mean ± standard deviation or median, as appropriate, while categorical variables were expressed as frequencies and percentages. As questionnaire score distributions were non-normal, nonparametric statistical tests were applied. Comparison of scores across SCARS severity categories was performed using the Kruskal-Wallis test. Correlation between SCARS, BICI, FASQoL, and Goodman-Baron scores was assessed using Spearman’s rank correlation coefficient. A p-value of <0.05 was considered statistically significant.

## Results

A total of 150 participants were included in the study. The majority of participants belonged to the 21-25-year age group (95/150; 63.3%), followed by the 26-30-year age group (27/150; 18.0%). Females constituted 69.3% (104/150) of the study population, while males accounted for 30.7% (46/150), yielding a male-to-female ratio of 1:2.26 (Table 1).

**Table 1:** Distribution of the study population across demographies (N=150)

Variable	Number of participants (n)	Percentage (%)
Age Group (Years)		
≤20	19	12.7
21–25	95	63.3
26–30	27	18
31–35	8	5.3
>35	1	0.7
Gender		
Female	104	69.3
Male	46	30.7

Regarding acne duration, 53 participants (35.3%) had acne for less than 1 year, whereas 49 (32.7%) reported a duration of 1 to 3 years. Acne duration exceeding 3 years was observed in 48 participants (32.0%). Scar duration of 1 to 3 years was noted in 63 participants (42.0%), followed by a duration between 6 months and 1 year in 39 participants (26.0%). The cheeks were the most commonly involved site, affecting 131 participants (87.3%), followed by the forehead in 57 (38.0%). Chin and nose involvement were observed in 24 (16.0%) and 23 (15.3%) participants, respectively. Mandibular and upper lip involvement was comparatively less frequent. With regard to prior treatment history, 70 participants (46.7%) had received topical therapy, 32 (21.3%) had received oral isotretinoin, and 9 (6.0%) had received oral antibiotics (Table 2).

**Table 2:** Distribution of the study population across clinical profiles (N=150)

Parameter	Number of participants (n) N = 150	Percentage (%)
Duration of acne		
< 1 year	53	35.3
1 to 3 years	49	32.7
>3 years	48	32
Duration of acne scars		
< 6 months	22	14.7
6 months – 1 year	39	26.0
1–3 years	63	42.0
> 3 years	26	17.3
Site of facial acne scars		
Cheeks	131	87.3
Forehead	57	38.0
Chin	24	16.0
Nose	23	15.3
Mandibular area	18	12.0
Upper lip	7	4.7
History of previous treatment		
Topical treatment only	70	46.7
No prior treatment	35	23.3
Oral isotretinoin	32	21.3
Oral antibiotics	9	6.0
Combination therapy / Others	4	2.7

**Figure 1:**
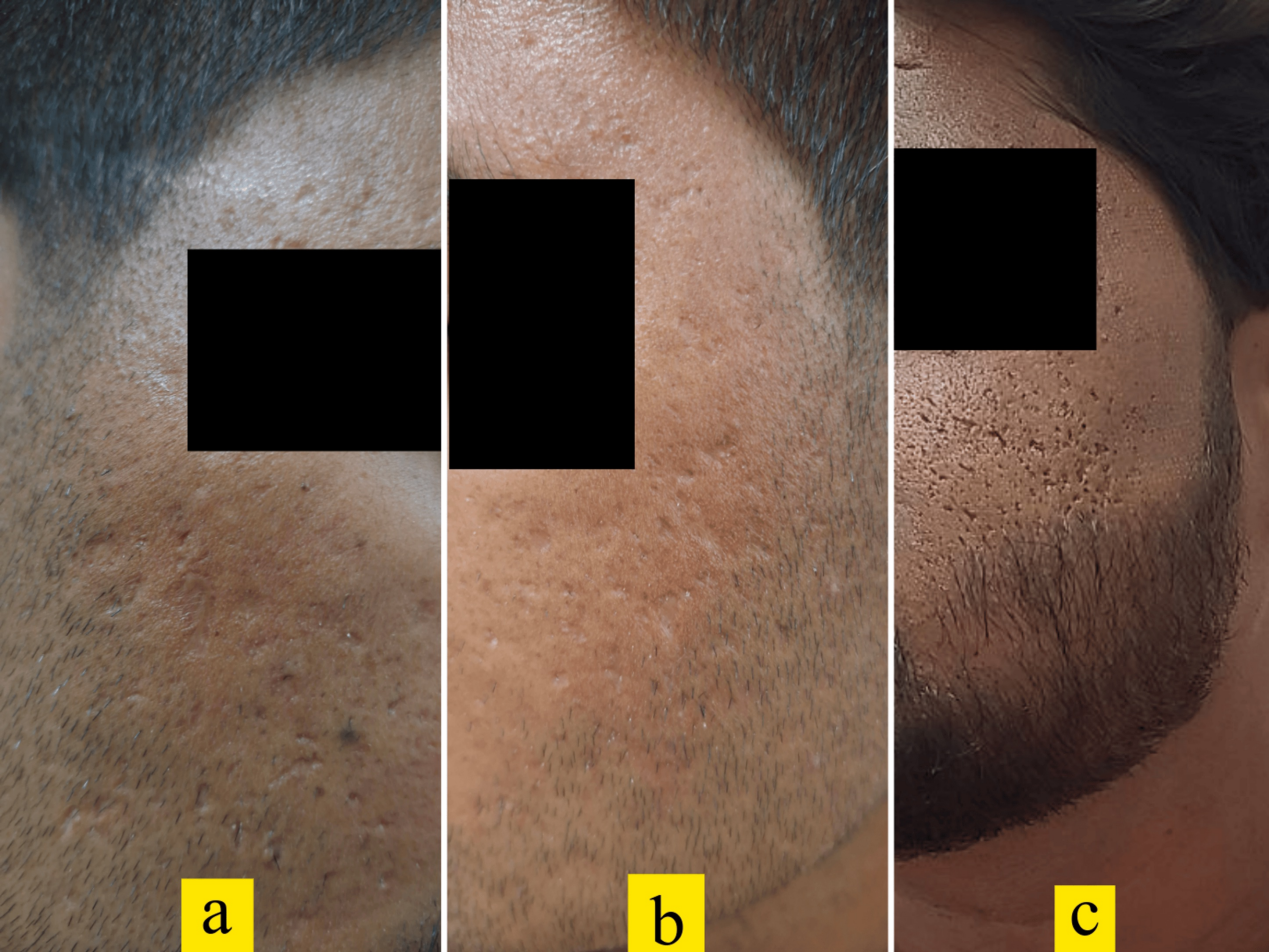
Different types of acne scars a: Boxcar scars; b: Rolling scars; c: Icepick scars

Mixed scar morphology was more common than single scar type. Icepick scars were the most frequently observed morphological subtypes (Figure 1), present in 118 participants (78.7%), followed by boxcar scars in 92 (61.3%) and rolling scars in 86 (57.3%). Hypertrophic scars were observed in 14 participants (9.3%). Mixed scar morphology was identified in 104 participants (69.3%) (Table 3).

**Table 3:** Distribution of study population across the morphology of acne scars (N=150)

Morphological type of acne scar	Number of participants (n)	Percentage (%)
Icepick scars	118	78.7
Boxcar scars	92	61.3
Rolling scars	86	57.3
Hypertrophic scars	14	9.3
Pattern of morphology		
Single morphology	46	30.7
Mixed morphology	104	69.3

Assessment using the SCARS questionnaire demonstrated that mild scarring was the most common category, observed in 54 participants (36.0%). Severe or very severe scarring was noted in 23 participants (15.3%). Selection of the highest severity score across individual SCARS items was relatively infrequent (Table 4).

**Table 4:** Item-wise distribution of SCARS questionnaire scores (N=150) SCARS: Self-Assessment of Clinical Acne-Related Scars Item 1 assessed the overall appearance of acne scars, Item 2 assessed scar depth, Item 3 assessed scar color, Item 4 assessed scar texture, and Item 5 assessed the extent of facial acne scarring.

SCARS Item	0, n (%)	1, n (%)	2, n (%)	3, n (%)	4, n (%)
Item 1	24 (16.0)	59 (39.3)	50 (33.3)	15 (10.0)	2 (1.3)
Item 2	40 (26.7)	56 (37.3)	46 (30.7)	8 (5.3)	0 (0.0)
Item 3	24 (16.0)	61 (40.7)	47 (31.3)	18 (12.0)	0 (0.0)
Item 4	37 (24.7)	60 (40.0)	44 (29.3)	9 (6.0)	0 (0.0)
Item 5	28 (18.7)	66 (44.0)	44 (29.3)	8 (5.3)	4 (2.7)

Based on the Goodman and Baron qualitative grading system, Grade III scarring was the most common category, observed in 80 participants (53.3%), followed by Grade IV scarring in 29 participants (19.3%).

The mean total FASQoL score was 6.79 ± 7.11, with scores ranging from 0 to 33. The majority of participants (120/150; 80.0%) had total FASQoL scores between 0 and 10, whereas 22/150 participants (14.7%) had scores between 11 and 20 (Table 5).

**Table 5:** Frequency distribution of FASQoL (N=150) FASQoL: Facial Acne Scar Quality of Life

Parameter	Value
0–10	120 (80.0%)
11–20	22 (14.7%)
21–30	7 (4.7%)
31–40	1 (0.7%)
Mean ± SD (Min, Max)	6.79 ± 7.11 (0, 33)

Comparison of BICI scores across SCARS severity categories demonstrated a statistically significant increase in mean BICI scores with increasing scar severity (Kruskal-Wallis test, p < 0.001). Similarly, FASQoL scores showed a statistically significant association with SCARS severity categories (Kruskal-Wallis test, p < 0.001) (Table 6).

**Table 6:** Association of BICI and FASQoL scores across SCARS severity categories (N=150) The Kruskal-Wallis test was used for comparison of BICI and FASQoL scores across SCARS severity categories. BICI: Body Image Concern Inventory; FASQoL: Facial Acne Scar Quality of Life; SCARS: Self-Assessment of Clinical Acne-Related Scars

SCARS Severity	n	Mean BICI Score ± SD	p-value	Mean FASQoL Score ± SD	p-value
Clear / Almost clear	28	32.32 ± 12.79		2.50 ± 4.58	
Mild	54	34.48 ± 11.74		5.26 ± 5.40	
Moderate	45	39.44 ± 12.39		8.13 ± 7.06	
Severe / Very severe	23	50.39 ± 13.17	<0.001	13.00 ± 8.59	<0.001
Total	150	38.01 ± 13.58	—	6.79 ± 7.11	—

**Figure 2:**
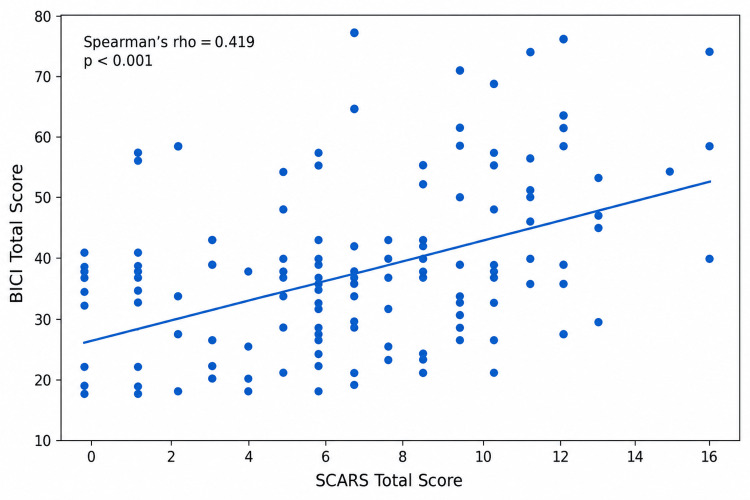
Correlation between Self-Assessment of Clinical Acne-Related Scars (SCARS) scores and Body Image Concern Inventory (BICI) scores among study participants (N=150)

**Figure 3:**
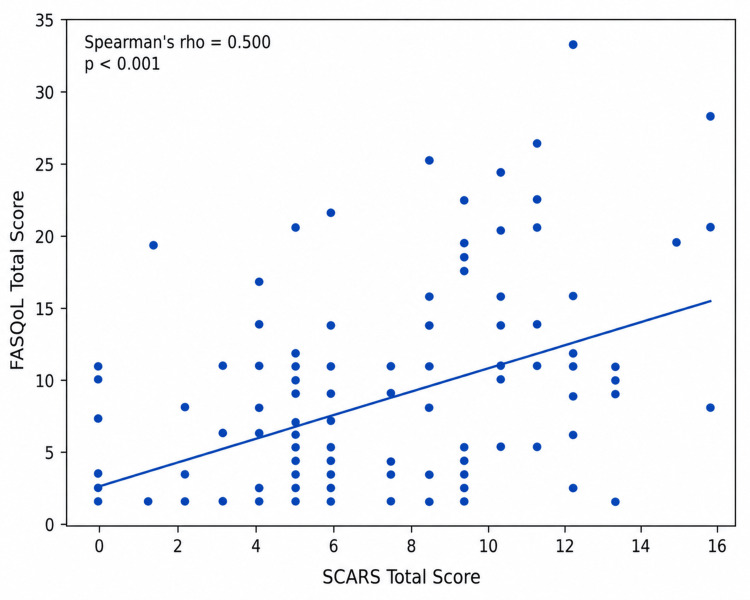
Correlation between Self-Assessment of Clinical Acne-Related Scars (SCARS) scores and Facial Acne Scar Quality of Life (FASQoL) scores among study participants (N=150)

**Figure 4:**
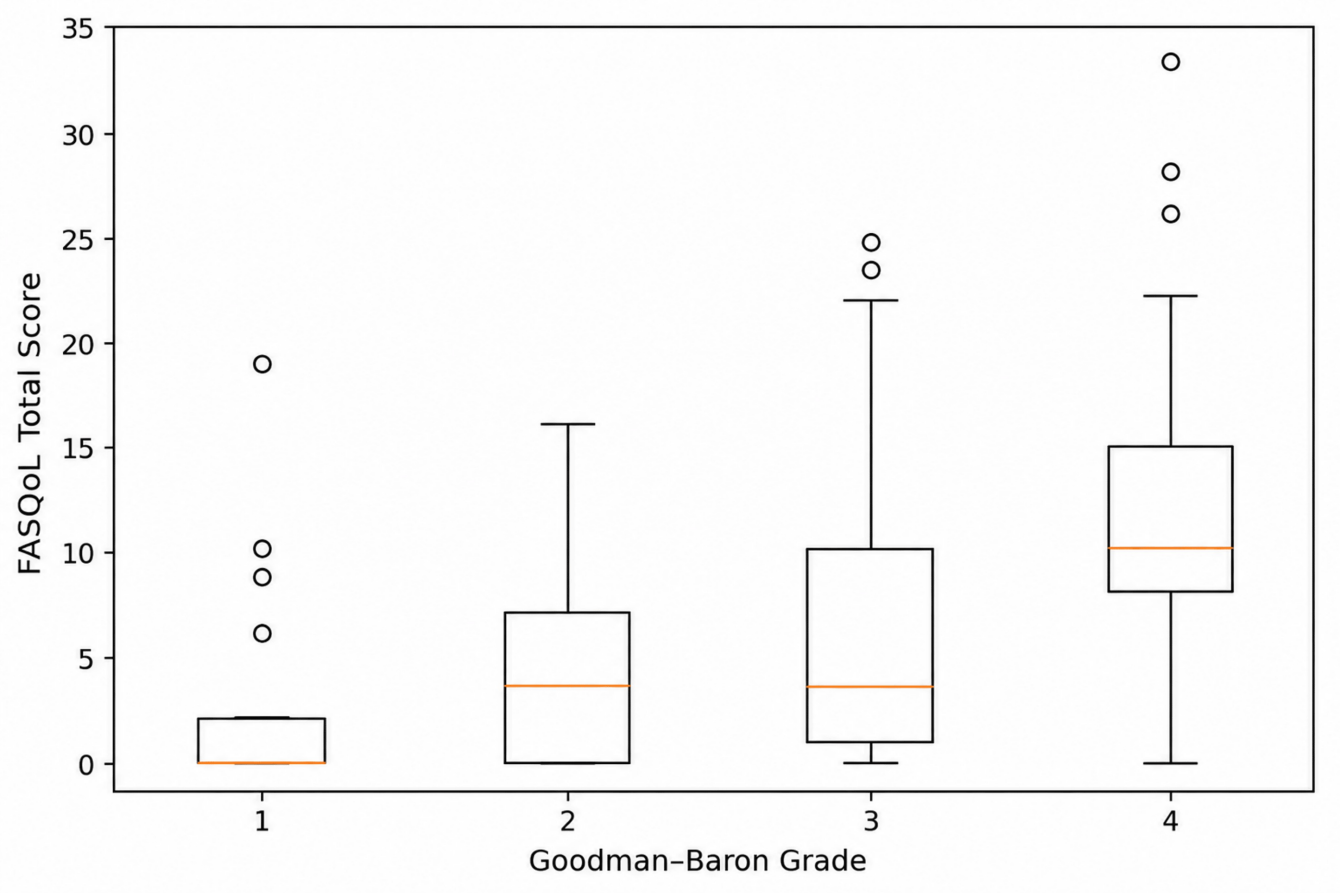
Distribution of Facial Acne Scar Quality of Life (FASQoL) scores across Goodman-Baron qualitative acne scar grades (N=150)

Spearman’s rank correlation analysis demonstrated a moderate positive correlation between SCARS and BICI scores (rₛ = 0.419, p < 0.001), as well as between SCARS and FASQoL scores (rₛ = 0.500, p < 0.001). Goodman-Baron grade also demonstrated a moderate positive correlation with FASQoL scores (rₛ = 0.448, p < 0.001) and BICI scores (rₛ = 0.367, p < 0.001) (Table 7; Figures 2-4).

**Table 7:** Correlation between SCARS, Goodman-Baron grade, BICI, and FASQoL scores (N = 150) SCARS: Self-Assessment of Clinical Acne-Related Scars; BICI: Body Image Concern Inventory; FASQoL: Facial Acne Scar Quality of Life

Variables Compared	Spearman’s rho (rₛ)	p-value
SCARS total score vs BICI total score	0.419	< 0.001
SCARS total score vs FASQoL total score	0.500	< 0.001
Goodman–Baron Grade vs FASQoL Total Score	0.448	<0.001
Goodman–Baron Grade vs BICI Total Score	0.367	<0.001

## Discussion

Demographic distribution

Although acne and acne scars are frequent dermatological disorders and the majority of patients explain that the changed texture of their skin has affected their psycho-social life, the strategic scientific studies conducted are very few. Thus, we conducted the present study to evaluate the association between clinician- and patient-perceived severity of acne with body image concerns and its correlation with facial acne scarring and quality of life, using validated standardized questionnaires.

In the present study, the majority of participants (63.3%; n = 95) belonged to the 21-25-year age group, with most individuals falling within the second and third decades of life, reflecting the higher incidences in young adults.

Similarly, Tan J et al., in their multi-country population-based survey among 723 adults, reported a mean age of approximately 24 years, with most participants in early adulthood [5]. Similarly, Heah MM et al., in a Malaysian cross-sectional study with 175 patients, observed that the majority of individuals with facial acne scars were aged between 20 and 30 years [17]. Indian data from Agrawal DA et al. also indicated that most patients presenting with post-acne scarring at their tertiary care settings were young adults [18].

The predominance of young adults in these studies aligns with the natural history of acne vulgaris, wherein inflammatory lesions peak during adolescence and scarring becomes clinically evident in subsequent years.

In the present study, females constituted 69.3% (n = 104) of the study population, while males comprised 30.7% (n = 46), yielding a male-to-female ratio of 1:2.26. Similarly, Tan J et al. reported that approximately 66% of participants of their cohort were females [5]. Heah MM et al. also documented a higher proportion of female in their cohort evaluating the psychosocial impact of acne scarring. These findings suggest that women may be more likely to seek medical attention for residual facial scarring [16]. However, Agrawal DA et al., in their hospital-based study from North India, reported a male predominance in post-acne scars [18].

This difference might explain the variations in study design, referral patterns of the study setting, and sociocultural influences across epidemiological regions. Thus, the difference in biological severity of acne by gender, societal, ethnicity-oriented, traditional practices, and geographical is needs further observational studies.

Duration of acne and acne scars

In the present study, 32.0% (n = 48) participants reported a duration of active acne exceeding >3 years, while 32.7% (n = 49) participants had acne for 1-3 years. Agrawal DA et al. reported that greater severity of active acne and the longer duration were significantly associated with higher grades of scarring [18]. Similarly, Heah MM et al. demonstrated that delayed initiation of acne treatment by more than a year was associated with increased odds of developing severe scars, with an observed odds ratio (OR) of 2.78.18. These findings collectively emphasized the role of sustained inflammation in the pathogenesis of permanent dermal remodeling.

Treatment history

In the present study, 46.7% (n = 70) received only topical therapy, 23.3% (n = 35) received no prior treatment, and only 21.3% (n = 32) received oral isotretinoin. Thus, nearly 70% of patients had either no treatment or topical-only therapy, which may indicate delayed escalation in moderate-to-severe acne. These findings indicate that a considerable proportion of patients either had delayed initiation of systemic therapy or had received suboptimal treatment before the development of scarring. Agrawal DA et al. found that patients not able to receive early and appropriate treatment for moderate-to-severe acne were more likely to develop higher grades of acne scarring (p < 0.05). In their study, oral isotretinoin showed significantly lower severity of scarring compared to oral antibiotics alone [18]. Similarly, Heah MM et al. reported that delayed treatment initiation was significantly associated with increased odds of severe scar formation [17].

Morphological profile of facial acne scars

In the present study, atrophic scars constituted the predominant morphological type. Icepick scars were noted among 78.7% (n = 118), boxcar scars in 61.3% (n = 92), rolling scars in 57.3% (n = 86), and hypertrophic scars among 9.3% (n = 14) study participants. Mixed morphological patterns accounted for 69.3% (n = 104), indicating that the majority of individuals exhibited more than one scar subtype. Agrawal DA et al. also reported multiple morphological patterns in their patients. Similar to our study population, the icepick scar pattern was the most common morphological type (94%), followed by rolling scars (86%) and boxcar scars (54%), with hypertrophic or keloidal scars observed in approximately 10% of their patients [18].

Tan J et al. methodologically focused on atrophic variants considering their previous clinical experience among outpatients, reinforcing that collagen loss rather than overproduction is the predominant pathological process in acne scarring [5].

The predominance of atrophic scars in the present study is consistent with the pathophysiological mechanism of acne scarring, wherein inflammatory destruction of dermal collagen leads to net tissue loss and surface depression. The high prevalence of mixed morphological patterns likely reflects repeated inflammatory episodes affecting different dermal depths over time, resulting in variable scar architecture within the same patient. The relatively low frequency of hypertrophic scars may be attributable to individual wound-healing characteristics and regional variations in fibrotic response.

Acne scar severity analysis (Goodman-Baron and SCARS)

In the present study, clinician-based assessment using the Goodman and Baron qualitative grading system demonstrated that the majority of participants (53.3%) had Grade III (moderate) scarring, followed by Grade IV (19.3%), Grade I (15.3%), and Grade II (12.0%). Similarly, patient-reported severity using the SCARS instrument showed that 36.0% of participants were categorized as mild, 30.0% as moderate, and 15.3% as severe/very severe.

Unlike our observation, Agrawal DA et al. reported that moderate to severe grades constituted a substantial proportion of patients presenting with post-acne scarring, with a significant association between severity of active acne and severity of scarring [18]. Tan J et al. demonstrated increasing self-perceived scar severity being significantly associated with worsening of psychosocial outcomes, highlighting the relevance of patient-reported severity in understanding disease burden [5]. Furthermore, Layton et al., during the development of the SCARS and FASQoL instruments, emphasized that patient-perceived severity may not always completely correspond to clinician-graded assessment, thereby underscoring the need for dual evaluation [15].

The overall concordance between the Goodman-Baron and SCARS categories indicates that structural deformity is generally recognized by patients. The findings support the clinical relevance of integrating clinician-based grading with patient-reported severity measures, as structural assessment alone may not fully capture the perceived burden of acne scarring.

Body image concern

In the present study, the mean BICI score was 38.01 ± 13.58, reflecting a moderate degree of appearance-related concern among participants with facial acne scars. A clear gradient was observed across SCARS severity categories, with mean BICI scores increasing progressively from the clear group (32.32) to the severe group (50.39), and the difference was statistically significant (Kruskal-Wallis H = 27.11, p < 0.001). The approximately 18-point difference between the clear and severe categories highlights a clinically meaningful gradient in appearance concern. Evidence from international research supports this observation. Tan J et al., in their large multi-country survey, demonstrated that increasing self-reported scar severity was associated with higher dysmorphic concern scores, with a subset of individuals exhibiting clinically significant appearance preoccupation [5]. Heah MM et al. also documented that patients with more severe scars experienced greater psychological distress, including anxiety and depressive symptoms, suggesting that visible facial scarring may heighten emotional vulnerability [17].

Mahajan S et al. reported significant body image dissatisfaction and the incidence of psychiatric illness having a positive correlation with acne scars [14]. The progressive increase in BICI scores across severity categories in the present study suggests that structural visibility and depth of scarring may intensify negative self- evaluation.

Quality of life impairment

The mean FASQoL score of our study population was 6.79 ± 7.11, indicating an overall mild-to-moderate scar-specific QoL impairment within the study population. The majority of participants (80%) had scores within the lower range (0-10); However, a progressive rise in FASQoL scores was observed across increasing SCARS severity categories: severe SCARS group mean: 13.00 ± 8.59, clear group mean: 2.50 ± 4.58. The average Goodman-Baron Grade and SCARS scores had a weak correlation with the FASQoL score (r 0.5 and 0.448, respectively; p <0.001).

Thus, severe cases had more than fivefold higher mean scores compared to clear cases, indicating marked functional burden. Findings from international literature reflect a similar severity-dependent pattern. Tan J et al., in their population-based study, reported a mean Dermatology Life Quality Index (DLQI) score of 6.26 and demonstrated that higher self-perceived scar severity was associated with greater impairment in emotional and social functioning. Their study further noted that even individuals with mild scarring reported measurable psychosocial burden [5]. In a cohort by Heah MM et al., patients with severe and very severe acne scars reported significant impairment in quality-of-life, with higher anxiety and depression scores [17].

Mahajan S et al. reported marked impairment in quality-of- life among patients with acne scars, with higher mean DLQI scores. They further noted significant associations between depressive symptoms and body image disturbance, suggesting that psychosocial dimensions may amplify functional limitation [14]. It should also be noted that patients with active acne lesions were not excluded from the present study if clinically significant acne scarring was present. Consequently, a proportion of the observed body image concerns and quality-of-life impairment may have been influenced by active acne itself in addition to the effects of acne scarring.

Relatively lower overall mean FASQoL scores observed in the present study compared to some hospital-based cohorts may reflect differences in severity distribution, sociocultural factors, treatment-seeking behavior, or other contextual influences within the study population.

Nevertheless, the clear stepwise increase in scores across severity categories suggests a significant association between perceived structural deformity and emotional distress and social discomfort. The persistence and visibility of facial scars may influence self-confidence in interpersonal interactions, particularly in socially interactive settings.

Limitations

The present study has several limitations. The cross-sectional study design precludes assessment of causal relationships between acne scar severity, body image concerns, and quality-of-life impairment. As the study was conducted at a single tertiary care center, generalizability to community-based populations and other sociocultural settings may be limited. Psychological assessment was performed using validated screening instruments without formal psychiatric diagnostic evaluation. Recall bias related to acne duration, treatment history, and onset of scarring may have influenced participant responses. Additionally, the absence of longitudinal follow-up limited assessment of temporal changes in psychosocial burden and post-treatment outcomes. Female participants constituted a larger proportion of the study population, which may limit the generalizability of the findings across genders. Patients with active acne lesions were not excluded if clinically significant acne scarring was present. Therefore, active acne itself may have contributed to body image concerns and quality-of-life impairment independent of acne scarring and could have acted as a potential confounding factor in the assessment of psychosocial outcomes.

Clinical implications

Facial acne scarring may exert clinically significant psychosocial effects beyond cosmetic disfigurement. Integration of patient-reported outcome measures alongside clinician-based scar grading may provide a more comprehensive assessment of acne scar burden. Early and appropriate treatment of inflammatory acne may help reduce permanent scarring and its associated psychosocial impact.

## Conclusions

Atrophic scars constituted the predominant morphological pattern, with icepick, boxcar, and rolling scars being the most frequently observed subtypes. Mixed scar morphology was common among the study population. Increasing acne scar severity demonstrated a significant association with greater body image concern and quality of life impairment. Participants with more severe scarring reported greater appearance-related distress, social self-consciousness, avoidance of social interactions, dissatisfaction with facial appearance, and increased concern regarding perceived facial defects. Scar-specific quality of life impairment was observed across emotional and social domains, including reduced self-confidence and discomfort in interpersonal interactions. These findings highlight the significant psychosocial burden associated with facial acne scarring beyond cosmetic disfigurement.
